# National Food, Nutrition, and Physical Activity Survey of the Portuguese General Population (2015-2016): Protocol for Design and Development

**DOI:** 10.2196/resprot.8990

**Published:** 2018-02-15

**Authors:** Carla Lopes, Duarte Torres, Andreia Oliveira, Milton Severo, Sofia Guiomar, Violeta Alarcão, Elisabete Ramos, Sara Rodrigues, Sofia Vilela, Luísa Oliveira, Jorge Mota, Pedro J Teixeira, Paulo J Nicola, Simão Soares, Lene Frost Andersen, The IAN-AF Consortium

**Affiliations:** ^1^ Department of Public Health and Forensic Sciences, and Medical Education, Faculty of Medicine University of Porto Porto Portugal; ^2^ Epidemiology Research Unit Institute of Public Health University of Porto Porto Portugal; ^3^ Faculty of Nutrition and Food Sciences University of Porto Porto Portugal; ^4^ National Health Institute Doutor Ricardo Jorge Lisbon Portugal; ^5^ Institute of Preventive Medicine and Public Health Faculty of Medicine University of Lisbon Lisbon Portugal; ^6^ Research Centre in Physical Activity, Health and Leisure Faculty of Sports University of Porto Porto Portugal; ^7^ Centro Interdisciplinar de Estudo da Performance Humana Faculty of Human Kinetics University of Lisbon Lisbon Portugal; ^8^ SilicoLife, Lda Braga Portugal; ^9^ Faculty of Medicine University of Oslo Oslo Norway; ^10^ University of Porto Porto Portugal

**Keywords:** surveys, nutritional surveys, exercise, public health, children, adults, elderly

## Abstract

**Background:**

The assessment of food consumption data using harmonized methodologies at the European level is fundamental to support the development of public policies. Portugal is one of the countries with the most outdated information on individual food consumption.

**Objective:**

The objective of this study was to describe the design and methodology of the National Food, Nutrition and Physical Activity Survey, 2015-2016, developed to collect national and regional data on dietary habits, physical activity (PA), and nutritional status, in a representative sample of the Portuguese general population (3 months-84 years).

**Methods:**

Participants were selected by multistage sampling, using the National Heath Registry as the sampling frame. Data collection, during 12 months, was harmonized according to European guidelines (EU-MENU, European Food Safety Authority [EFSA]). Computer-assisted personal interviewing (CAPI) was performed on a specific electronic platform synchronized with nutritional composition data and considering the FoodEx2 classification system. Dietary assessment was performed using 24-hour recalls (two nonconsecutive, 8-15 days apart) or food diaries in the case of children aged <10 years, complemented with a food propensity questionnaire; PA data (International Physical Activity Questionnaire [IPAQ], the Activity Choice Index [ACI], and 4-days PA diaries); sociodemographic data, and other health-related data were also collected.

**Results:**

A sample of 6553 individuals completed the first interview, and 5811 participants completed two dietary assessments. The participation rate among eligible individuals was 33.38% (6553/19,635), considering the first interview, and 29.60% (5811/19,635) for the participants with two completed interviews (about 40% in children and adolescents and 20% in elderly individuals). Results of the survey will be disseminated in national and international scientific journals during 2018-2019.

**Conclusions:**

The survey will assist policy planning and management of national and European health programs on the improvement of nutritional status and risk assessment related to food hazards, and the enhancement of PA. The infrastructures and data driven from this Survey are a solid basis to the development of a future national surveillance system on diet, PA, and other health behaviors reproducible over time.

## Introduction

Monitoring food consumption at the national level is imperative to assist health policy making, to provide a solid basis for the development of nutritional and food security policies, and to plan future research. According to the European Report on Food Consumption Survey Methods (EFCOSUM) [1], Portugal is one of the European countries with the most outdated information on individual food habits. The first national dietary survey was conducted in 1980 [2], and since then no nationwide individual information has been collected using harmonized instruments capable of answering official European indicators.

The European Food Safety Authority (EFSA) has conducted the Pan-European Survey “What's in European menu?” (EU-Menu), promoting the development and testing of harmonized instruments and protocols for evaluating food consumption across Europe [3], in which Portugal has been involved by conducting the National Food, Nutrition and Physical Activity Survey in 2015-2016 (acronym: IAN-AF 2015-2016).

This paper aims to describe the design and data collection methodologies used in the IAN-AF 2015-2016. The survey aimed to collect nationwide data (from 3 months to 84 years of age) on dietary habits (foods, nutrients, dietary supplements, food safety, and insecurity), physical activity (PA) (sedentary behaviors, sports, and active choices in daily living) and their relation with health determinants, namely, socioeconomic factors.

## Methods

### Sampling

A probabilistic sample of the Portuguese general population aged between 3 months and 84 years was selected by multistage sampling, using the National Health Registry (RNU coding) as the sampling frame. Participation was independent of the regular attendance to the National Health System.

The first step of sampling was based on the random selection of primary health care units, stratified by the 7 Statistical Geographic Units of Portugal (NUTS II), weighted by the number of individuals registered in each health unit. The second step of sampling was based on the random selection of registered individuals in each health unit, according to sex and age groups.

The sample selection was performed in consecutive recruitment waves to use the most updated versions of the National Health Registry lists (4 recruitment waves for infants and toddlers and 2 recruitment waves for the remaining age groups). Individuals with the following criteria were excluded: (1) living in collective residences or institutions (eg, elderly in retirement homes or individuals in hospitals, at prisons, or military barracks); (2) living in Portugal for less than 1 year (nonapplicable to infants); (3) non-Portuguese speakers; (4) with diminished physical and/or cognitive abilities that hamper participation (eg, blind, deaf, with diagnosed dementias); and (5) deceased.

Individuals with no established contact after all planned attempts were considered with unknown eligibility. Eligible participants without availability for the 2 interviews during the evaluation period or who missed appointments were classified as eligible nonparticipants. In addition, for eligible participants aged 65 years or more, a screening of cognitive impairment was performed by using the Mini-Mental State Examination test [4]. The classification of cognitive impairment was performed using the scale’s score according to the education level [5,6]: illiterate and 15 points or less; 1 to 11 years of education and 22 points or less; and at least 11 years of education and 27 points or less. For these individuals only few socio-demographic and anthropometrics information were collected.

### Estimated Sample Size

The sample size was estimated by assuming a mean population energy intake of 2000 kcal/day (standard deviation, SD=500) and an effect size of 8%, with a confidence level of 95%. The sample size for each geographical region was estimated in 603 individuals (a total of 4221 individuals in the 7 regions).

To estimate the study design effect, the following information was taken into consideration: (1) a coefficient of variation of cluster sizes defined as 0.4; (2) data from cluster-based studies with primary health care setting in Portugal, measuring the dependency effect of exposures such as body mass index or energy intake (mean intracluster correlation coefficient of 58%); and (3) a mean number of participants reachable in each primary health care unit, depending on the field work management (30 individuals were estimated to be evaluated during 4 weeks). Considering these data, a design effect of 1.20 (an increase of 20% of the sample size) was estimated.

As a result of the settled design effect, the number of individuals to be assessed in each region was estimated as 724, resulting in a total sample of 5068 individuals in the 7 regions. Thus, taking into consideration the distribution of the Portuguese population according to the Census 2011 [7], the sample size required to have representativeness at the national level was of 5068 individuals: 935 children and adolescents (0-17 years), 3262 adults (18-64 years), and 871 elderly (65-84 years). To accomplish the EFSA requirements of including 260 individuals in each age group (130 by sex), an oversampling of children less than 1 year (3-11 months) (6 times the initial proportion) and 1 to 2 years (3 times the initial proportion) was considered, followed by a redistribution of the sample size for the other age groups. In each health unit, the number of individuals to be assessed by sex and age group was fixed.

An additional sample of pregnant women was also estimated (n=200) using the same sampling frame, resulting in 2 to 3 pregnant women by each health unit. A potential participation rate of 70% in the first interview and in the second interview was defined, resulting in 50% (70%×70%=49%) of nonresponse, unreachable individuals, incomplete questionnaires, and drop-offs expected. Thereby, the number of participants to be selected and contacted was estimated to be 10,204 (5102 x 2). After a pilot study, a more conservative participation rate of 20% (5102 x 5=25,510) was assumed.

**Figure 1:**
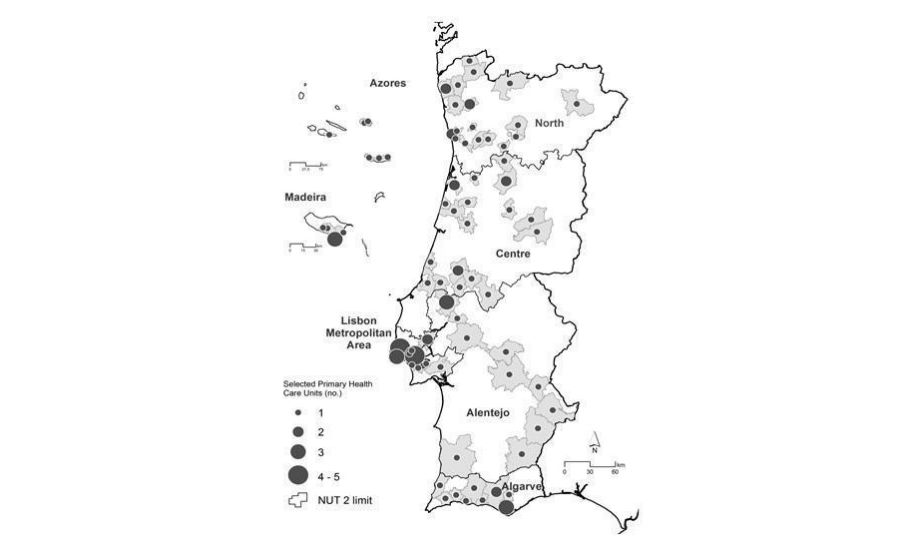
Spatial distribution of the primary health care units, weighted by the number of registered individuals: the IAN-AF 2015-2016 survey (NUT: Statistical Geographic Units of Portugal).

Assuming a one-month period for data collection in each health unit, and an estimation of approximately 30 participants by unit, the number of health units needed to be selected was 21 by region. This number was applied to the North, Centre, and Lisbon regions, but due to logistic constraints related to field work efficiency and the low number of health units in the other regions, 12 health units were selected in Alentejo and Algarve, and 6 health units were selected in Madeira and Azores. Figure 1 shows the spatial distribution of the health units, randomly selected in each of the 7 regions (NUTS II), weighted by the number of registered individuals.

### Field Work Management and Recruitment

The field work management team included a national coordinator, a subnational coordinator, and 5 regional coordinators. The field work team included 40 interviewers with nutrition or dietetics background and one statistician.

All the procedures related to the management of the field work and participants’ recruitment were performed through an electronic platform (“You eAT&Move”) based on a client-server software architecture, which was specially developed for the survey purposes.

Participants were contacted by telephone to check their willingness to participate. At maximum, 6 contact attempts in different daytimes and day hours were performed. If participants did not answer after 4 attempts, a short message was sent, followed by more 2 contacts attempts, before they were classified as unknown eligibility. If an oral acceptance was provided by individuals, an invitation letter with participation details was sent by post. If individuals refused to participate, some questions included in a short refusal questionnaire were collected. The examination location was selected according to participant’s preference. One of the following two options was provided: the participant’s home or the health units they belong to.

### Data Collection

Overall, the survey includes the evaluation of the following dimensions: (1) dietary and nutritional intake, (2) eating habits and behaviors, (3) dietary and nutritional supplements use, (4) food insecurity, (5) PA and sedentary behaviors, (6) sociodemographics, (7) general health data, (8) anthropometrics, and (9) biochemical indicators of nutritional intake (subsample).

Most of the procedures of data collection were adapted from the EFSA Guidance in view of the EU Menu methodology [3]. Data were collected by trained fieldworkers using computer-assisted personal interviewing (CAPI), during 12 months (October 2015-September 2016), distributed over the 4 seasons and including all days of the week to incorporate seasonal effects and day-to-day variation in food intake. For interviews, the “You eAT&Move” software includes the following 3 main modules:

*You* module, that includes sociodemographic questions, anthropometric measures, general health data, food consumption by a food propensity questionnaire (FPQ), and food insecurity

*eAT24* module, which allows the collection and description of food consumption data by 24-hour recalls (or food diaries) with food pictures for portion size estimation, synchronized with nutritional composition data of foods and recipes

*Move* module, which allows the collection of PA data and includes the IPAQ questionnaire, the Activity Choice Index (ACI), and PA diaries, synchronized with metabolic equivalents data associated with each type of PA.

Table 1 shows the overview of data dimensions collected in the IAN-AF 2015-2016 survey, by first and second interviews and age groups (plus pregnant women).

### Dietary Assessment Methods

Dietary intake was obtained by 2 nonconsecutive one-day food diaries for children aged less than 10 years and 2 nonconsecutive 24-hour recalls for the remaining age groups. The time between interviews was set at 8 to 15 days. The days of reporting were randomly selected, but participants were able to change them according to their own availability for the interview. For Saturdays, a 24-hour recall was performed on Mondays. For adolescents from 10 to 14 years, the 24-hour recall was administered with the presence of one of the parents or other main caregiver; for adolescents from 15 to 17 years, the 24-hour recall was administered without the need of parents’ help. For children aged less than 10 years, the 2 nonconsecutive one-day food diaries were followed by a face-to-face interview, allowing the parent or other main caregiver to add details related to food description and quantification.

To validate nutritional intake in participants aged 18 years or more, data from the 24-hour recall were compared against urinary biomarkers. In a subsample of adults (n=94), 24-hour urinary samples were collected during the day before the second interview. The urinary concentration of sodium, potassium, iodine, and total nitrogen were assessed.

### The eAT24 Module

Dietary intake data were collected using the “eAT24” module (electronic assessment tool for 24-hour recall), which allows the assessment of dietary data by an automated multiple-pass method for 24-hour (5 steps) [8]. All foods, including beverages and dietary supplements consumed during a 24-hour period, were recorded per eating occasion and quantified and described as eaten.

The eAT24 methodology requires the description of consumed foods during the dietary interview through several facets and respective descriptors, using the FoodEx2 classification system [9]. The place and time of meal consumption are also recorded for each eating occasion.

The software allows subsequent conversion of foods into nutrients, using by default the Portuguese food composition table [10]. Several activities were performed for updating or developing the food-related lists and the classification and description of foods. The initial food list was based on the 962 food items from the Portuguese food composition table updated with several other food items, such as infant formula, baby foods, and ready-to-eat items (such as desserts and drinks), resulting in a list of 2479 food items and 117 dietary supplement items. A recipe module was also created including 1696 recipes. In this module, recipes were disaggregated into raw ingredients allowing the description and quantification of each item. Recipes were described as 5 facets (production method, brand, preservation method, packaging material, and reheating method) and subsequently disaggregated into their ingredients. Recipe ingredients were also described according to the FoodEx2 classification system [9]. Nutritional composition of recipes is calculated according to the methodology proposed by the European Food Information Resource (EUROFIR) network of excellence “Proposal for the harmonization of recipe calculation procedures” [11]. The software allows the inclusion of a new food item or a new recipe during the data collection process.

Different methods are available for use for food and recipe quantification: (1) weight or volume method, (2) standard unit method, (3) photo method (food picture book including 186 food photo series [with 6 portions each food/recipe], and 11 household measures photo series), (4) household measure method, and (5) default portion method.

### The Food Propensity Questionnaire

A FPQ was also used for usual intake modeling purposes [3], adapted from a previous version proposed by the “Pilot study in the view of a Pan European dietary survey” (PANEU project) [12]. The main objective is to identify never-consumers, to minimize problems with the day-to-day variation of intake that might affect the estimation of usual intake, typical of single or few short-term measurements (such as 24-hour recalls) [13]. The FPQ includes a general list of food and beverage groups and a list of specific foods and beverages important for risk assessment. The number of items in the list and the reference period of consumption differ by age group: children <9 years—45 food items, reference period: the last month; adolescents and adults—49 food items, reference period: the last 12 months; and pregnant women—49 food items, reference period: the last 3 months.

**Table 1:** Overview of data dimensions collected in the National Food, Nutrition and Physical Activity Survey, 2015-2016, (IAN-AF) by age groups (plus pregnant women).

Modules and dimensions		Age groups (years)								Pregnant women
		3 months-2	3-5	6-9	10-14	15-17	18-64	65-84		
**You**										
	Sociodemographics (SD)^a^	SD1	SD1	SD1	SD2	SD2	SD3	SD3	SD3	
	General health (G)^b^	G1	G2	G2	G3	G3	G4	G4	G5	
	Eating behaviors (EB)^c^	EB1	EB2	EB2	EB2	EB2	EB3	EB3	EB3	
	Anthropometrics (A)^d^	A1	A2	A2	A2	A2	A2	A2	A3	
	Household food security (HFS)	-	-	-	-	-	HFS	HFS	HFS	
	Food Propensity Questionnaire (FPQ)^e^	-	FPQ1	FPQ1	FPQ2	FPQ2	FPQ2	FPQ2	FPQ3	
**eAT24**										
	Food diaries 1 (FD1)^f^ and 2 (FD2)	FD1	FD2	FD2	-	-	-	-	-	
	24-h recall 1 (24R)	-	-	-	24R	24R	24R	24R	24R	
	24-h recall 2 (24R)	-	-	-	24R	24R	24R	24R	24R	
**Move**										
	Physical activity diaries (PAD)	-	-	PAD	PAD	-	-	-	-	
	International Physical Activity Questionnaire (IPAQ)	-	-	-	-	IPAQ	IPAQ	IPAQ	IPAQ	
	Activity Choice Index (ACI)					ACI	ACI	ACI	ACI	
	Sedentary behaviors (SB)^g^	-	SB1	SB2	SB3	SB4	SB5	SB5	SB6	

^a^SD1 and SD2 differ from SD3 by including also questions on country of origin, nationality, professional activity, education of parents, and the current school year (in SD2 only). These versions do not have questions on marital status and household income (only included in SD3).

^b^G1 assesses previous diseases with need of regular medical care (predefined list of diseases) and identifies the health professional assistant. G2 only assesses previous diseases with need of regular medical care. G3 assesses previous diseases with need of regular medical care, and also the general health condition and current and past smoking habits. G4 evaluates previous diseases with need of regular medical care, and also diseases previously diagnosed by a physician, the general health condition, current and past smoking habits, and gynecological history in women. G5 assesses the general health condition before and during pregnancy, smoking habits before and during pregnancy, gynecological history, and data on current pregnancy (gestational weeks, health problems, etc).

^c^EA1 include questions relating to breastfeeding, consumption of different milk options, food diversification, and a brief food frequency questionnaire (FFQ) of interest items. EA2 only includes a fruit and vegetables FFQ. EA3 include besides a fruit and vegetables FFQ, questions about organic foods consumption, food safety, and a salt consumption scale.

^d^A1 includes weight and length driven from the child health booklet and measured weight and height. A2 includes self-reported weight and height and measured weight, height, and waist and hip circumferences. A3 does not include waist and hip circumference measurements, and evaluates self-reported height and weight before pregnancy.

^e^FPQ1 assesses the consumption of 45 food items in the last month. FPQ2 assesses the consumption of 49 food items (including alcoholic beverages) in the last 12 months, and an option for seasonal consumption is available. FPQ3 is similar to FPQ2, but the reference period is the last 3 months.

^f^FD1 differs from FD2 because it has a specific structure for registering breastfeeding and formula feeding.

^g^All SB include information about sleep habits on weekdays and weekend days and questions about regular and programmed PA. SB1 differs from SB2 and SB3 on the type of sedentary behaviors asked. SB4 and SB5 include also a question about physical or sedentary choices.

### Dietary Supplements

The use of dietary supplements was retrieved by 2 methods. The first method used the 24-hour recall, in which dietary supplements and foods consumed during a 24-hour period were recorded per consumption occasion and quantified and described as consumed. Supplements were described according to 6 facets (supplement source, target group, place of acquisition, packaging material, brand, and physical state). This interview-based dietary assessment instrument allows a very detailed description of supplements consumed in the course of the preceding day.

The second method asked the frequency of use, in the last 12 months, of a predefined list of 16 different dietary supplement types (eg, supplements of vitamins, such as C, D, and folate; supplements of minerals, such as calcium and iron; supplements of multivitamins; supplements of fatty acids, herbs, plants, and probiotics).

### Food Insecurity

Food insecurity [14] assessment was obtained by applying a slightly modified questionnaire developed by Radimer et al [15], widely applied in the evaluation and monitoring of public food assistance programs in the United States and in other countries, and by Bickel [16], which was adapted for Portugal by INSA and ERS/USDA. The 3-stage design questionnaire keeps respondent burden to the minimum needed to get reliable data. It provides estimates of food insecurity for households with or without children under the age of 18 years by collecting information on 4 underlying dimensions and experience of food insecurity: availability, access, utilization, and stability or resilience. In this way, food insecurity is associated not only with structural poverty but also with transitional—but not less serious—conditions of scarce, in particular, financial resources. The food insecurity status of each household lies somewhere along a continuum, extending into the following 3 categories: food security (households had no problems or anxiety about consistently accessing adequate food), moderate food insecurity (households reduced the quality, variety, and desirability of their diets, but the quantity of food intake and normal eating patterns were not substantially disrupted), and severe food insecurity (at times, eating patterns of one or more household members were disrupted and food intake was reduced because the household lacked money and other resources for food during the year).

### Physical Activity Assessment Methods

For children (6-9 years) and adolescents (10-14 years), PA was assessed by diaries (2 consecutive days during the week and 2 of the weekend), and for the other age groups (≥15 years), the assessment methods included the International Physical Activity Questionnaire (IPAQ) short version [17] and the ACI [18].

The PA diaries were an adaptation of a model proposed by Bouchard et al [19] in which children registered their activities in a logbook for each 15 min interval during 4 days, according to previous written instructions.

Additional questions on sedentary behaviors were also asked in all age groups (including children aged 3-6 years). From the age of 15, self-reported activities representative of “opportunistic” active choices in daily routine during the last month (eg, taking the stairs, parking further away from an entrance, or choosing to stand instead of sitting) were assessed with 6 item response options on a 5-point Likert scale (ie, 1=never, 5=always), by using the ACI scale [18]. Composite scales can be calculated by averaging the items. A question on structured leisure time PA activity, detailing the type and time of activity, was also asked in all age groups from 6 years of age.

The collection of PA data was performed using the e-module “Move,” including the IPAQ questionnaire and PA diaries, synchronized with metabolic equivalents data associated with each type of PA. For all types of activity, daily energy expenditure was computed using the energy cost of each activity as estimated from reference values for participants aged 15 years or more [20,21] and using an adaption for children based on values proposed by Ridley et al [22].

In children, energy expenditure was estimated by multiplying the related MET by the self-reported time spent in each activity (min/day) recorded in the diary. Individual daily energy expenditure was computed as the mean expenditure of the 4-day diaries. To validate the information from PA diaries, PA was objectively measured by accelerometry in a subsample of 35 participants from 6 to 14 years. Participants were asked in the first interview to wear an accelerometer (ActiGraph GT3X models; Pensacola, FL) during 4 days, including 2 consecutive weekdays and 2 consecutive weekend days, the same registered in the PA diary.

### Anthropometrics

Adolescents and adults were asked to self-report their actual height and weight before the performance of objective measurements. In children, this information was retrieved from their health booklets, the last registry was considered. In pregnant women, weight and height was reported before pregnancy.

Anthropometric measurements, including length/height, weight, and body circumferences, were performed in both children and adults according to standard procedures [23] by trained observers. Height was measured to the nearest centimeter, with participants in a standing position with light clothing and barefoot, using a portable wall stadiometer (SECA 213, Hamburg, Germany). For participants whose height was not possible to measure, the hand length was measured using a pocket ruler as an alternative [24]. For children under 2 years, recumbent length was measured to the nearest 0.1 cm with a measuring rod with large calipers (SECA 207; Hamburg, Germany).

Body weight was measured in the same conditions to the nearest tenth of a kilogram using a digital scale (SECA 813, Hamburg, Germany). For children under 2 years, a specific pediatric digital weight scale was used (SECA 354, Hamburg, Germany), and measurements were conducted with participants naked and without diaper, to the nearest 0.01 cm.

Arm, waist, and hip circumferences were measured, using an anthropometric tape, in all age groups except in children under 3 years and in pregnant women. Arm circumference was measured at the marked level of the mid-acromiale-radiale. Waist circumference was measured at the level of the narrowest point between the lower costal border and the iliac crest. Hip circumference was measured at the level of greatest posterior protuberance of the buttocks. All these circumferences were performed to the nearest 0.1 cm.

As part of quality control procedures, a bubble level was used to check the best position for the equipment in the room; a small platform was used to allow the direct observation of values from the stadiometer and the calibration of scales using standard weights of 5000 g and 500 g and their combinations was performed.

### Quality Control

Several quality control actions were undertaken before, during, and after the fieldwork process, including the following: (1) testing the e-platform in several pilot studies performed across the different geographical regions, (2) recruitment of interviewers with thorough knowledge of the available foods on the market and traditional recipes, (3) on-going training of those interviewers (distance electronic devices were used to assist interviews, when needed), and (4) control of individual energy and macronutrient intake at the end of interview, and definition of a maximum food weight to easily identify information biases. This control is directly integrated in the software. For example, for the total energy intake, a minimum of 122 Kcal or 500 Kcal and a maximum of 2816 Kcal or 4000 Kcal were considered according to the age groups 3 months to 9 years or 10 years to 84 years, respectively. An outlier is signalized with an alert message allowing the interviewer to perform the corrections during the interview. Few other quality control actions that were undertaken are as follows: (1) preliminary statistical analysis during fieldwork to check possible information bias, the distribution of interviews by days of the week and seasons, (2) registry of doubts in an editor book to be solved by the research team, (3) calibration of anthropometric devices each 3 months, (4) application of a refusal questionnaire to nonresponders to check the representativeness of the final sample, (5) identification of under- and over-reporters of energy intake [3,25], outliers, and removal of intra-individual variability of dietary intake.

### Ethics

Ethical approval was obtained from the National Commission for Data Protection, the Ethical Committee of the Institute of Public Health of the University of Porto, and the Ethical Commissions of each one of the Regional Administrations of Health.

All participants were asked to provide their written informed consent according to the Ethical Principles for Medical Research involving human subjects expressed in the Declaration of Helsinki and the national legislation. Written agreements from the parents were required for children and adolescents below 18 years. Adolescents (10-17 years) were also asked to sign the consent form together with their legal representative.

All documents with identification data were treated separately and stored in a different dataset.

## Results

### Sample Size

A total of 5819 participants completed 2 interviews (5811 with two complete dietary assessments) and 6553 completed only the first interview (Table 2). Approximately 23% are children under the age of 10 years, 11% are adolescents (10-17 years), 53% are adults (18-64 years), and 13% are elderly (65-84 years). An additional sample of 184 pregnant women was evaluated.

**Table 2:** Final sample size by sex and age groups—the National Food, Nutrition and Physical Activity Survey, 2015-2016, (IAN-AF).

Age groups		Children (<10 years)			Adolescents (10-17 years)			Adults (18-64 years)			Elderly (≥65 years)			Total
		Male	Female	Male		Female	Male		Female	Male		Female		
Selected participants, n		1923	1965	952		988	8336		9384	3094		2541	29,183	
Unknown eligibility, n		388	404	197		163	1677		1960	458		369	5616	
Known eligibility, n		1535	1561	755		825	6659		7424	2636		2172	23,567	
Eligible, n		1410	1422	658		719	5725		5971	2037		1693	19,635	
Noneligible, n		125	139	97		106	934		1453	599		479	3932	
Contact rate, n (%)^a^		1410 (78.42)	1422 (77.88)	658 (76.9)		719 (81.5)	5725 (77.34)		5971 (75.29)	2037 (81.64)		1693 (82.10)	19,635 (77.76)	
**Participants’ 1st interview**														
	Cooperation rate, n (%)^b^	769 (54.54)	745 (52.39)	351 (53.3)		349 (48.5)	1881 (32.86)		1563 (26.18)	429 (21.06)		466 (27.53)	6553 (33.37)	
	Participation rate, n (%)^c^	769 (42.77)	745 (40.80)	351 (41.1)		349 (39.6)	1881 (25.41)		1563 (19.71)	429 (17.19)		466 (22.60)	6553 (25.95)	
**Participants with 2 dietary assessments**														
	Cooperation rate, n (%)^b^	667 (47.30)	660 (46.41)	319 (48.5)		313 (43.5)	1674 (29.24)		1428 (23.92)	358 (17.57)		392 (23.15)	5811 (29.60)	
	Participation rate, n (%)^c^	667 (37.10)	660 (36.14)	319 (37.3)		313 (35.5)	1674 (22.62)		1428 (18.01)	358 (14.35)		392 (19.01)	5811 (23.01)	

^a^Contact rate: eligible/(eligible + unknown eligible individuals).

^b^Cooperation rate: participants/eligible individuals.

^c^Participation rate: participants/(eligible + unknown eligible individuals).

The contact rate was 77.76%. The cooperation rate among eligible individuals was 33.37%, considering the first interview, and 29.60% for the participants with 2 completed dietary assessments. Similarly, the participation rate was 25.95% and 23.01%, respectively. The participation rates were higher in children and adolescents (approximately 40%) and much lower in the elderly (approximately 20%) (Table 2).

The characteristics of participants were compared with characteristics of individuals who refused to participate and who accepted to fill out a small refusal questionnaire by phone.

Information on some important indicators, such as sex, age, and region of residence; frequency of consumption of fruit and vegetables; regular practice of leisure-time PA; and self-reported weight and height; were available. Individuals who refused to participate were older (over 65 years: 22% vs 13%) and less educated (over 12 years: 19% vs 27%), although for variables representing the main areas of the survey—fruit and vegetables consumption (≥5 portions/day: 18.6% vs 18.1%), practice of regular leisure-time PA (33% vs 39%), and obesity (12.4% vs 12.7%)—the differences are of a small magnitude.

### Data Analysis

All statistics that will be used to calculate future estimates driven from this survey, both at national or regional levels, will include the weighting of the sample data. The weighted sample represents the number of individuals from the national general population that are represented by each individual in the study sample. The sample weighting includes the following: (1) initial weights to overcome the different probability of sampling units selection; (2) a second weight to overcome the different probability of individuals selection in each unit, by sex and age (considering the total population, by sex and age groups in the closest recruitment wave); and (3) correction of these initial weights for nonresponse bias.

The distribution of the interviews by week days and seasons was also analyzed. The distribution of interviews by day of the week was 18.3%, 20.5%, 18.5%, 16.9%, 8.2%, 5.1%, and 12.6% from Monday to Sunday, respectively. The distribution of the interviews by season showed that spring was the season where most interviews were performed (35.9%), followed by winter (28.3%), summer (24.7%), and finally by autumn (11.1%).

Final databases were already checked and ready for analysis. Specific statistical analysis for identification of outliers and removal of intra-individual variability preceded the final analysis. Results of the survey will be disseminated in national and international scientific journals during 2018-2019.

## Discussion

### Strengths and Limitations

The response rates were lower than expected, particularly among adults and elderly individuals, despite several dissemination activities (eg, through regional media) that were undertaken to promote the survey close to the population. However, results were similar to other national European dietary surveys using the same sampling approach [26,27]. Participants in the survey were older and less educated than those who refused to participate but did not differ on the prevalence of fruit and vegetables consumption, practice of regular leisure-time PA, and obesity.

The used sampling frame covers the entire population resident with a national identification card, which means that illegal residents such as refuges or irregular immigrants are not included. However, most of these individuals, even if included in the survey by the sampling strategy, would be noneligible, since they are non-Portuguese speakers (considered as an exclusion criteria). Moreover, the proportion of legal foreign residents is only 3.7% [7]. Some other vulnerable and marginalized population groups, such as homeless people, despite not having participated, represent a small part of the Portuguese population (696 according to Census 2011) [7]. The absence of these individuals in the sample is not expected to influence the final results and the representativeness of the sample.

The distribution of interview week days and seasonality was checked and, although not exactly as planned, it follows the minimum requisites of having a considerable number of registries in all the week days and seasons. Fewer registries on Saturdays result from the fact that no interviews were scheduled on Sundays, as the report of both Saturdays and Sundays was conducted on Mondays. Friday also had fewer registries than other weekdays because not all health care units were opened on Saturdays, despite the continuous efforts of the fieldwork team to have alternative spaces for the interviews on Saturdays. Additionally, autumn was the season with the lowest proportion of interviews, because it coincided with the beginning and the end of the field work, when there are less concentrated interviews.

Following the European standards of dietary assessment is a major strength of this project as it will allow the comparison of important indicators in Europe in several different domains of food and nutrient intake, eating behaviors, nutritional status, food safety, and food insecurity. Information of the major contributors for sugar, sodium, or fat intake will orient new community interventions. The information could also give support to the assessment of the impact of current legislative measures, such as those related to the reduction of sugar in soft drinks or reduction of salt in bread. Furthermore, accurate and detailed food consumption data are important for the assessment of risk exposure to potentially hazardous substances. The use of the Foodex2 food classification system, proposed by EFSA [9], improves the possibility of getting information of risk assessment related to food biological and chemical hazards, namely, main exposures to chemicals added intentionally to food such as additives (eg, artificial sweeteners, preservatives, and artificial colors) and to chemical compounds from food processing (eg, nitrosamines, heterocyclic amines, and aromatic hydrocarbons) and packaging materials (eg, bisphenol A and phthalates) that enter the food supply inadvertently.

For PA, the harmonization of procedures in Europe is still under discussion, but having information on different domains such as overall activity level, structured leisure-time physical exercise, and sedentary behaviors could be useful for supporting the estimation of indicators and to better develop new population interventions.

### Future Perspectives

Findings from this survey will allow having national updated knowledge on the distribution of diet, PA, and other health-related risks according to sex, age, education, and geographical region. It also serves as an important descriptive starting point for future follow-up surveys in specific target groups. It expects to contribute to the development of national and European evidence-based policies that translate research into effective nutrition and health strategies, sustainable over time. A comprehensive analysis according to socioeconomic dimensions will also contribute to the development of policies with impact in equity and human well-being.

It is expected that the structure and information driven from this survey could also contribute to develop and consolidate solid infrastructures for epidemiological and public health research by building a future national functioning surveillance system on diet, PA, and other health behaviors, reproducible over time.

## Abbreviations

ACI: Activity Choice Index
CAPI: computer-assisted personal interviewing
eAT24: electronic assessment tool for 24-hour recall
EB: eating behaviors
EFSA: European Food Safety Authority
FD: food diary
FPQ: Food Propensity Questionnaire
G: general health
HFS: household food security
IAN-AF: The National Food, Nutrition and Physical Activity Survey, 2015-2016 (Portuguese acronym)
IPAQ: International Physical Activity Questionnaire
PA: physical activity
PAD: physical activity diaries
SB: sedentary behaviors
SD: sociodemographics
24R: 24-h recall
